# Inorganic Nitrogen Derived from Foraging Honey Bees Could Have Adaptive Benefits for the Plants They Visit

**DOI:** 10.1371/journal.pone.0070591

**Published:** 2013-07-29

**Authors:** Archana Mishra, Ohad Afik, Miguel L. Cabrera, Keith S. Delaplane, Jason E. Mowrer

**Affiliations:** 1 Department of Crop & Soil Sciences, the University of Georgia, Athens, Georgia, United States of America; 2 Department of Entomology, the University of Georgia, Athens, Georgia, United States of America; Helmholtz Centre for Environmental Research – UFZ, Germany

## Abstract

In most terrestrial ecosystems, nitrogen (N) is the most limiting nutrient for plant growth. Honey bees may help alleviate this limitation because their feces (frass) have high concentration of organic nitrogen that may decompose in soil and provide inorganic N to plants. However, information on soil N processes associated with bee frass is not available. The objectives of this work were to 1) estimate the amount of bee frass produced by a honey bee colony and 2) evaluate nitrogen mineralization and ammonia volatilization from bee frass when surface applied or incorporated into soil. Two cage studies were conducted to estimate the amount of frass produced by a 5000-bee colony, and three laboratory studies were carried out in which bee frass, surface-applied or incorporated into soil, was incubated at 25^o^C for 15 to 45 days. The average rate of bee frass production by a 5,000-bee colony was estimated at 2.27 to 2.69 g N month^−1^. Nitrogen mineralization from bee frass during 30 days released 20% of the organic N when bee frass was surface applied and 34% when frass was incorporated into the soil. Volatilized NH_3_ corresponded to 1% or less of total N. The potential amount of inorganic N released to the soil by a typical colony of 20,000 bees foraging in an area similar to that of the experimental cages (3.24 m^2^) was estimated at 0.62 to 0.74 g N m^−2^ month^−1^ which may be significant at a community scale in terms of soil microbial activity and plant growth. Thus, the deposition of available N by foraging bees could have adaptive benefits for the plants they visit, a collateral benefit deriving from the primary activity of pollination.

## Introduction

In most terrestrial ecosystems, nitrogen (N) is the most limiting nutrient for plant growth as evidenced by studies showing that addition of inorganic N increases plant productivity [1]. Mechanisms of N limitation include N fixation by clays, competition by microorganisms, reduced N diffusion due to low soil water content, and reduced N mineralization from organic residues due to low N content, acid pH, or low soil temperature and water content [2]. Insects may help alleviate this N limitation by reintroducing N into soil through their feces (frass). Most terrestrial insects are believed to excrete waste N as uric acid [3], [4], [5], but some insects such as the cotton stainer (*Dysdercus fasciatus*) excrete waste N as allantoin, which is a byproduct of uric acid decomposition [6]. Ammonium (NH_4_^+^) and urea also occur in frass but in comparatively low amounts [7], [5]. Decomposition of insect frass through the activity of heterotrophic microorganisms (bacteria and fungi) may lead to net N mineralization or net N immobilization depending on frass composition. Nitrogen mineralization is the conversion of organic N to NH_4_^+^, whereas N immobilization is the conversion of inorganic N (NH_4_^+^ or NO_3_^−^) to organic N. In a field study, Frost and Hunter [8] saw an increase in soil NH_4_^+^ as a result of decomposition of frass from the eastern tent caterpillar (*Malacosoma americanum*). Similarly, Zaady et al. [9] documented net N mineralization from termite (*Anacanthothermes ubachi* Navas) feces incubated in the dark at 20 to 25^o^C. In contrast, Lovett and Ruesink [10], working with gypsy moth frass (*Lymantria dispar*), and Kagata and Ohgushi [11], working with cabbage armyworm frass (*Mamestra brassicae* L.), found that frass decomposition led to N immobilization during 5 to 13 weeks of incubation.

When net N mineralization occurs, NH_4_^+^ is the first inorganic N species produced, which may be subsequently converted to nitrate (NO_3_^−^) through the process of nitrification [12]. The NH_4_^+^ produced through mineralization can also be lost to the atmosphere as ammonia gas (NH_3_), a process called ammonia volatilization [13]. Both inorganic N species (NH_4_^+^ and NO_3_^−^) are available for plant uptake, but some plants have preference of one N species over the other depending on stage of growth, soil pH, and other environmental factors. In general, plants adapted to low pH and reducing conditions usually take up NH_4_^+^, whereas plants adapted to higher pH and oxidizing conditions take up NO_3_^−^ [14]. Plants such as cereals, corn, sugar beets, and many grasses use either form of N, whereas plants such as tobacco, tomato, and potato, prefer a high NO_3_^−^/NH_4_^+^ ratio [12].

The frass of honey bees (*Apis mellifera* L.) has been used in studies of microbiological properties [15] and nitrogen composition [16] but no information is available on soil N transformations. Therefore, the objectives of this study were to: 1) estimate the amount of bee frass produced by a honey bee colony, and 2) evaluate N mineralization and NH_3_ volatilization from honey bee frass when surface applied or incorporated into soil. To accomplish these objectives, we conducted two field studies and three laboratory studies.

## Materials and Methods

No specific permits were required for the described field studies because they were located in general areas of a research farm owned by the University of Georgia. The location is not privately-owned or protected in any way, and the field studies did not involve endangered or protected species.

### Frass Production

Two cage studies were conducted to estimate the amount of bee frass produced by a honey bee colony. In the first study, two cages (1.8 m ×1.8 m ×1.8 m) were established on May 24, 2010, at the Horticulture Farm of the University of Georgia. The bottom of each cage contained Cecil loamy sand soil (fine, Kaolinitic, thermic Typic Kanhapludults) with bermudagrass (*Cynodon dactylon* L.) and tall fescue (*Lolium arundinacea* Schreb.) plants growing on it. One honey bee colony was introduced into each of the cages. Each colony included 500 g of bees (about 5000 individuals), two honey combs, and two brood combs. The bees were supplied with water and pollen patties during the experiment (25 g pure pollen from GloryBee Foods, Eugene, OR, mixed with 25 g sugar powder and 10 g honey). The amount of bee frass being deposited in each cage was estimated on eight separate dates from June 5 through June 27 by placing two sheets of corrugated plastic (0.1 m^2^) inside each cage for 24 or 48 hours. One sheet was placed in one corner of the cage and one sheet was placed at the center of the cage. The frass collected on the plastic sheets was dried at room temperature (23°C) for 24 hours, weighed, and analyzed as described below. The second study, established on August 21, 2010, was similar to the first except that there were four cages and each cage contained only bermudagrass plants. The amount of bee frass deposited in each cage was estimated on six dates from August 30 through September 21, as described above. The bee frass collected in these studies was used for the incubation studies after it was analyzed as described below.

Frass samples were ground and analyzed for pH, total C, total N [17], total P, and other elements (ICP-OES after acid digestion) (Tables 1 and 2). In addition, 1 g frass was extracted with 40 mL 1 M KCl and the extract was analyzed for NO_3_^−^ [18], NH_4_^+^ [19], urea [20], and uric acid. Uric acid content was determined by extracting 1 g frass with 500 mL 0.12 M sodium acetate in a water bath at 50°C for 2 hours. An aliquot of the extract was filtered directly into a 2-mL target vial for HPLC analysis using a Luer-type syringe with a 0.45-μm polypropylene filter. Analysis was performed on a Hewlett-Packard series 1100 HPLC instrument with degasser and 0.05 M KH_2_PO_4_ as mobile phase (prepared from HPLC-grade salt). Separation was achieved using an ODS-2 Hypersil LC column (250×4.6 mm; 5 μm particle sizes, and a UV/VIS detector (290 nm) was used to quantify the extracted uric acid as it eluted.

**Table 1:** pH, buffering capacity, total C, total N, and inorganic and organic N forms in soil and bee frass.

Material	pH	H^+^ Buffering Capacity	Total C	Total N	NH_4_^+^-N	NO_3_^−^-N	Uric Acid-N	Urea-N
	mmol H^+^kg^−1^pH^−1^		g kg^−1^		mg kg^−1^			
Soil	6.3	5	16.5	1.3	21	16	ND	ND
Frass	5.7	ND	531.5	51.5	624	10	11,400	202

ND  =  not determined.

**Table 2:** Selected elemental composition of soil and bee frass.

Material	P	Ca	K	Cu	Mg	S	Fe	Zn	B
	mg kg^−1^								
Soil	415	669	553	<0.4	251	238	10,203	11	<0.8
Bee Frass	7,228	3,465	11,476	7	1,947	2,689	794	61	68

### Incubation Studies

Three incubation studies were conducted to evaluate N mineralization and NH_3_ volatilization when bee frass is applied to soil. Studies 1 and 2 were conducted with surface-applied bee frass, whereas Study 3 was carried out with bee frass incorporated (mixed) into the soil. The soil used was collected from the upper 15 cm of an area mapped as Cecil loamy sand (fine, Kaolinitic, thermic Typic Kanhapludults) in the Georgia Piedmont (USA). The soil sample was air dried and passed through a 2-mm sieve before use. Soil characteristics included 0.203 g silt g^−1^, 0.037 g clay g^−1^, and 0.760 g sand g^−1^. Additional characteristics are presented in Tables 1 and 2. Soil pH was measured with a combination pH electrode in a 1∶1 (soil: deionized water) ratio. To determine the H^+^ buffering capacity of the soil, 20 g soil was mixed with 20 mL 0.01 M CaCl_2_, a pH measurement was made, and then 2.7 mL 0.023 M Ca(OH)_2_ was added and allowed to react for 30 min before a second pH measurement was made. Soil was analyzed for particle size by the pipette method [21]. Total C and N were analyzed by dry combustion [17] and total P and other elements by ICP-OES after acid digestion.

#### Study 1

The experimental units were 50-mL beakers (4 cm ID), each containing 20 g (dry weight equivalent) of soil and receiving one of the following treatments: 1) Soil without bee frass +2 mL deionized (DI) water, 2) Soil +2 mL DI water + bee frass surface-applied (20 mg) following water application, or 3) Soil + bee frass surface-applied (20 mg) prior to water application +2 mL DI water. The amount of DI water added was intended to bring the soil water content to 0.10 g H_2_O g^−1^ (approximately field capacity), and the timing of addition of DI water was intended to simulate rain before or after surface deposition of bee frass. The rate of frass application corresponded to 16 g m^−2^ which is the approximate amount of bee frass deposited by a colony of 5,000 bees in one month (see results of frass production below). Each experimental unit was placed into a 4-L glass jar containing 100 mL DI water at the bottom to minimize desiccation and an acid trap with 20 mL 0.25 M H_2_SO_4_ to trap volatilized NH_3._ Treatments were replicated five times and arranged in a completely randomized design inside an incubator set at 25°C for 15 days. Acid traps were changed every 3 days and analyzed for NH_4_^+^ to determine volatilized NH_3._ At the end of the study, the soil in each experimental unit was extracted with 160 mL 1 M KCl by shaking in a reciprocating shaker set at 120 oscillations per minute for 30 min. The extracts were analyzed for NH_4_^+^ and NO_3_^−^ as described above.

#### Study 2

The experimental units and treatments were the same as those described in Study 1, but this study was conducted for 45 days at 25°C with destructive extraction of four replicates of each treatment at 15, 30, and 45 days of incubation as described above. In addition to NH_3_ traps, the 4-L glass jars holding the experimental units also contained a beaker with 20 mL 0.2 M NaOH to trap carbon dioxide (CO_2_). These CO_2_ traps were changed every 3 days (as the NH_3_ traps) and were titrated to pH 7 with HCl after adding excess BaCl_2_.

#### Study 3

The experimental units were the same as those described in Study 1, but the treatments were as follows: 1) Soil + 20 mL DI water and 2) Soil +20 mL DI water + bee frass (20 mg) incorporated into the soil. Treatments were arranged in a completely randomized design inside an incubator set 25°C, and destructive extraction of four replicates of each treatment was carried out at 15 and 30 days of incubation as described above.

### Statistical Analysis

Cumulative net N mineralized and NH_3_ volatilized from the bee frass was calculated by subtracting inorganic N and volatilized NH_3_ in controls without bee frass addition. Data of net N mineralized and NH_3_ volatilized in the incubation studies were analyzed as a completely randomized design using PROC ANOVA in SAS [22]. Data of cumulative CO_2_ emission at different times were analyzed as a completely randomized design with repeated measures using PROC MIXED in SAS [22] and compound symmetry as covariance structure. Fisher's protected LSD was used to separate treatments means, and effects were considered significant when p<0.05.

## Results and Discussion

### Frass Production

The average daily amount of frass collected in the first study was 540±120 mg m^−2^ d^−1^ (95% confidence interval) when 5,000 bees foraged in an area of 3.24 m^2^. Thus, the monthly amount of frass deposited can be estimated at 16.2±3.6 g frass m^−2^ month^−1^, which is equivalent to 0.83±0.18 g N m^−2^ month^−1^ (assuming total N = 51.5 g kg^−1^, Table 1). In the second cage study, the average daily amount of frass was 450±170 mg frass m^−2^ d^−1^ (95% confidence interval). Using similar calculations as for the first study, the amount of frass deposited in the second study can be estimated at 13.5±5.1 g frass m^−2^ month^−1^, which is equivalent to 0.70±0.26 g N m^−2^ month^−1^. Taking into consideration the area of the cages (3.24 m^2^), the total amount of frass N produced by 5,000 bees in one month can be estimated at 2.69±0.58 g N month^−1^ in the first study and 2.27±0.84 g N month^−1^ in the second study. The significance of these N depositions is discussed below taking into account results of the incubation studies.

The bee frass collected from cages and used in our laboratory studies had a high total N content (51.5 g kg^−1^, Table 1) compared to results by McNally et al. [16] who found values ranging from 23.2 to 40.0 g N kg^−1^. In contrast, our bee frass had a smaller amount of uric acid (22% of total N) than the frass analyzed by McNally et al. (48 to 96% of total N), and the ratio of ammoniacal-N (NH_4_^+^ + NH_3_) to uric acid-N was low (0.055). In addition, frass pH was 5.7, indicating that the ammoniacal N was mainly present as NH_4_^+^. Nitrate (0.02% of total N) and urea (0.4% of total N) were present in low amounts. These analyses show that 99% of the N in frass was present in organic form. The C∶N ratio of the frass was 10.3, which indicated that frass decomposition would be expected to result in net N mineralization. In addition to C and N, bee frass contained significant amounts of P, K, Ca, Mg, and S (Table 2). Thus, frass decomposition may be important in recycling not only N but also other plant nutrients.

### Incubation Studies

#### Study 1

In the first surface-applied study, there was no difference between water-timing treatments (frass added before or after simulated rain) in the percentage of organic N mineralized from bee frass in 15 days (24%, Table 3). This value was similar to the percentage of uric acid-N in the organic N fraction of the frass (22%), which suggests that uric acid may have been the main source of mineralized N. Decomposition of uric acid through the uricase enzyme produces urea, which in turn is hydrolyzed by the urease enzyme to produce NH_4_^+^and CO_2_ [23]. The hydrolysis of urea consumes H^+^, which leads to a pH increase, its magnitude depending on initial pH, reaction rate, and H^+^ buffering capacity of the soil [24].
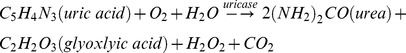
(1)

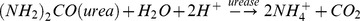
(2)

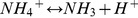
(3)


**Table 3:** Nitrogen released (initial inorganic N + mineralized N), N mineralized, and NH_3_ volatilized from bee frass in two surface-applied studies and one incorporated study held at 25°C for 15 to 45 days.

Study	Days	Treatment	Nitrogen Released	Nitrogen Mineralized	NH_3_ Volatilized
			% of Total N	% of Organic N	% of Total N
1	15	Water+Frass(surface)‡	25.9a†	25.0a	1.0a
1	15	Frass(surface)+Water	24.2a	23.2a	0.6a
					
2	15	Water+Frass(surface)	−3.1b	−4.3b	0.1a
2	15	Frass(surface)+Water	19.3a	18.3a	0.1a
					
2	30	Water+Frass(surface)	19.2a	18.3a	0.2a
2	30	Frass(surface)+Water	22.8a	22.0a	0.2a
					
2	45	Water+Frass(surface)	18.9a	17.9a	0.2a
2	45	Frass(surface)+Water	24.2a	23.5a	0.2a
					
3	15 & 30	Frass (Incorporated)	34.8	34.2	–

† Within a column and study period, means followed by the same letter are not significantly different according to Fisher's protected.

LSD at p<0.05.

‡ Water+Frass(surface)  = 2 mL of water added to 20 g of soil, then 20 mg of frass applied on the surface.

Frass(surface)+Water  = 20 mg of frass applied on the surface of 20 g of soil, then 2 mL of water added to soil.

Frass (Incorporated)  = 20 mg frass incorporated into 20 g soil, then 2 mL of water added to soil.

As pH at the site of urea hydrolysis increases, NH_4_^+^ converts to NH_3_ which in turn may be evolved from the soil in gaseous form [13]. In this study, the addition of DI water before or after applying frass did not affect the amount of volatilized NH_3_ (average  = 0.8% of the total N applied, Table 3). The low amount of NH_3_ loss agrees with results from a study with gypsy moth frass in which volatilized NH_3_ was 0.1% of total N in the frass [25]. The low amount of volatilized NH_3_ was probably due to the relatively low pH values of the frass (5.7) and soil (6.3) coupled to the H^+^ buffering capacity of the soil (5 mmol H^+^ kg^−1^ pH^−1^) and frass. If the entire mineralized N is assumed to have been derived from the conversion of uric acid to urea, which in turn was subsequently hydrolyzed in the upper 2 mm of soil, then using the measured buffering capacity of the soil it is possible to estimate an increase in pH of 0.1 units. Such a small pH increase from 6.3 to 6.4 would lead to minimal NH_3_ losses as observed in this study.

There was no difference between water-timing treatments in the amount of inorganic N released (initial inorganic N + mineralized N) from bee frass in 15 days (average of 25% of applied N), but NO_3_-N expressed as percentage of total inorganic N released ((NH_4_^+^+NO_3_^−^)-N) was 22% in the treatment that received water before bee frass and 65% in the treatment that received water after the frass. These results suggest that bee frass may have contained water-soluble compounds that partially inhibited nitrification. When water was added after bee frass these compounds may have been leached into the soil, thereby eliminating this partial inhibition.

#### Study 2

In the second surface-applied study, there was net N immobilization at 15 days in the treatment that received DI water before applying bee frass (Table 3). This suggests that the bee frass contained labile organic compounds whose decomposition led to N immobilization as has been observed in gypsy moth frass [10]. In contrast, when DI water was added after applying bee frass, 18% of the organic N was mineralized in 15 days. The addition of water after frass caused a fast colonization by fungi that was evident by extensive hyphal development on the soil surface. This fungal development may have been responsible for the observed N mineralization. In addition, water added after the frass may have leached into the soil those compounds that caused N immobilization in the treatment where water was added before the frass.

**Figure 1:**
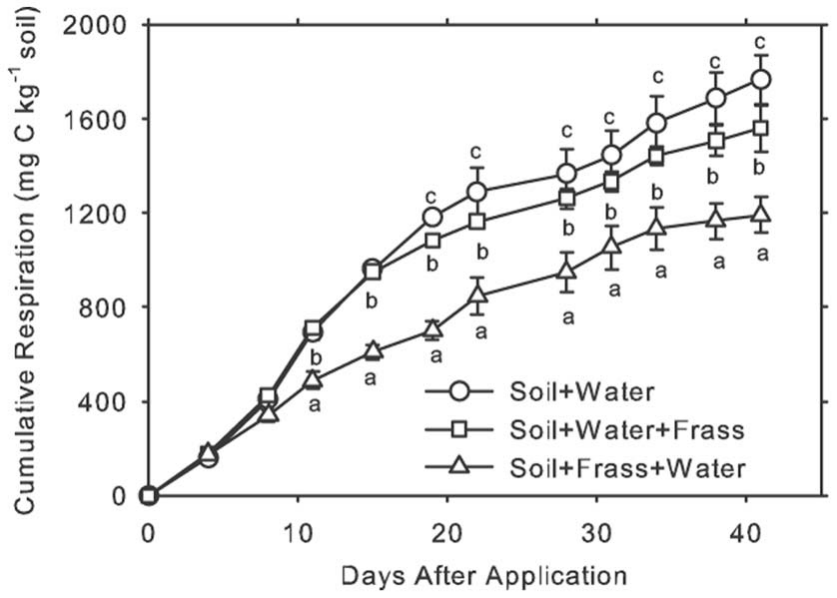
Cumulative respiration from three treatments in Study 2. The following treatments were incubated at 25°C for 45 days: 1) Soil+Water  =  soil (20 g) without bee frass; 2) Soil+Water+Frass  =  soil (20 g) with simulated rain (2 mL) before surface application of bee frass (20 mg); 3) Soil+Frass+Water  =  soil (20 g) with simulated rain (2 mL) after surface application of bee frass (20 mg). Bars are standard deviations.

It is interesting to note that the total CO_2_ emission was lower in treatments with frass than in the control treatment (Fig 1). This suggests that compounds present in the frass decreased soil respiration. Furthermore, starting on day 13, cumulative CO_2_ emission was lower in the treatment where DI water was added after the frass when compared to the other treatments (Fig. 1). This effect may have been caused by the added water leaching inhibiting compounds from the frass into the soil.

In spite of the differences observed between treatments on day 15, at 30 and 45 days of incubation both treatments showed net N mineralized averaging 20% of the organic N. As in the first study, this value is similar to the percentage of uric acid in the organic N fraction of the frass. Also, in agreement with results from the first study, the total amount of NH_3_ volatilized was minimal in both treatments (0.2% of applied N, Table 3). At 30 and 45 days of incubation, there was no difference between water-timing treatments in the amount of inorganic N released from bee frass (average of 21% of applied N, Table 3). In both treatments, more than 90% of the released inorganic N was in the form of NO_3_^−^, which indicates that conditions were adequate for nitrification.

#### Study 3

In this third study in which bee frass was incorporated into the soil, the amount of organic N mineralized and the amount of inorganic N released from bee frass was the same at 15 and 30 days of incubation (Table 3). However, the amount of NO_3_-N released expressed as percentage of total inorganic N was 63% at 15 days and 98% at 30 days. These results indicate that nitrification did not proceed as fast as N mineralization. The percentage of organic N mineralized (34%) was greater than the percentage of uric acid in the organic N fraction, which indicates that incorporating bee frass into the soil allowed decomposition of additional N-containing organic compounds. These compounds may have been products of uric acid decomposition such as allantoin and amino acids [6].

In general, our results of net N mineralization seem to agree with results by Frost and Hunter [8] who saw an increase in NH_4_^+^ in soil as a result of decomposition of eastern tent caterpillar (*Malacosoma americanum*) frass. In contrast, our general results differ from other studies that have found net N immobilization. For example, Kagata and Ohgushi [11] observed net N immobilization when incubating cabagge armyworm (*Mamestra brassicae L*.) frass (C∶N = 3.2 to 4.9) with soil for five weeks. Similarly, Lovett and Ruesink [10] incubated gypsy moth (*Lymantria dispar*) frass (C∶N = 20) with soil for 120 days and observed N immobilization during the first 90 days, with most of the N immobilized during the first 10 days. Our bee frass had a C∶N ratio of 10 (intermediate between the studies referenced above) and led to net N mineralization. Thus, as is the case for crop residues [26], C∶N ratio by itself may not be sufficient to determine whether a given frass will mineralize or immobilize N. Our results suggest that uric acid content in bee frass may be a good indicator of mineralizable N when bee frass is surface applied. Gordillo and Cabrera [27] found that uric acid is also a good indicator of mineralizable N in poultry litter. Thus, using bee frass composition to estimate mineralizable N may be a useful approach because the composition of bee frass varies with bee diet [16]. Additional studies should be conducted with different bee frass samples to determine if uric acid content could be used to estimate mineralizable N.

### Significance of Inorganic N released

The total amount of frass N (organic + inorganic N) deposited by 5,000 bees in one month was estimated at 2.27 to 2.69 g N month^−1^. Assuming that the average inorganic N released from bee frass is similar to the average value observed in the incubation studies (22% of total N), the amount of inorganic N released from the bee frass of 5,000 bees in one month would be 0.50 to 0.59 g inorganic N month^−1^. This amount of N was derived from colonies with about 5,000 bees, but an average bee colony may have 20,000 bees in spring [28] and therefore may result in a monthly release of inorganic N of about 2.0 to 2.4 g inorganic N month^−1^. Foragers can fly several kilometers from the nest, but the most common distance is 600–800 m [29]. More significantly, owing to the celebrated group recruitment behavior of honey bees [30], the concentration of foragers can for a period of days be local and intense – even to the level of patches or individual plants (ie., a scale congruent to the cages used in our study). The area and amount of time in which such concentrated bee foraging occurs is a product of diverse factors including bloom phenology, bloom density, pollinator populations, and weather, but if it is assumed that concentrated foraging by 20,000 bees occurs during one month in an area similar to that of the cages (3.24 m^2^), this would result in a monthly release of inorganic N of 0.62 to 0.74 g N m^−2^ month^−1^ which can be expected to exceed the N deposition otherwise realized from non-concentrated background deposition of insects or other fauna.

Inorganic N release at this scale may have system-level impacts in terms of soil microbial activity and plant growth for the plant communities within the foraging range of a honey bee colony. The estimated amount of inorganic N released (0.62 to 0.74 g inorganic N m^−2^ month^−1^) is within the range of inorganic N mineralized from soil organic matter in Coastal Plain soils. For example, Egerkraut et al. [31] found that the amount of N mineralized from the upper 120 cm of a Norfolk soil in southern Georgia (USA) ranged from 2.6 to 6.7 g inorganic N m^2^ in 4 months, which corresponds to 0.65 to 1.7 g inorganic N m^2^ per month. Thus, concentrated bee foraging could provide an amount of inorganic N similar to that released from soil organic matter in similar Coastal Plain soils, and therefore may have ecological significance. It should be noted, however, that where concentrated bee foraging does not occur, the amount of inorganic N released from deposited bee frass is not likely to be significant for plant growth.

Our results raise the fascinating question whether this ephemeral, albeit intense deposition of available N could have adaptive benefits for the plants visited by bees, a collateral benefit deriving from the primary activity of pollination. A similar collateral benefit was noted when the flight activity of honey bees was shown to interrupt feeding by herbivorous caterpillars [32]. These kinds of indirect interactions between members of food webs warrant more attention to elucidate their evolutionary and community-regulating significance.

## Conclusions

The bee frass used in our studies had about 99% of total N in organic form with 22% of the organic N as uric acid and 0.4% as urea. Mineralization of bee frass N during 30 days at 25°C released 20% of the organic N as inorganic N when bee frass was surface applied and 34% when frass was incorporated into the soil. Our results suggest that the main source of mineralizable N was the uric acid present in the frass. Ammonia volatilization losses corresponded to 1% or less of total N. To our knowledge, this is the first study that evaluated N mineralization and NH_3_ volatilization from bee frass. The potential amount of inorganic N released from a typical colony of 20,000 bees foraging in a small area (3.24 m^2^) was estimated at 0.62 to 0.74 g N inorganic m^−2^ month^−1^, which may be significant at a community scale in terms of soil microbial activity and plant growth. Thus, the deposition of plant-available N by foraging bees could have adaptive benefits for the plants they visit, a collateral benefit deriving from the primary activity of pollination. This is an example of many kinds of indirect interactions between members of food webs that may have important but poorly understood regulating effects on ecological communities.
